# Associations of KLOTHO-VS heterozygosity and α-Klotho protein with cerebrospinal fluid Alzheimer's disease biomarkers

**DOI:** 10.1177/13872877251326199

**Published:** 2025-03-20

**Authors:** Alzbeta Katonova, Ross Andel, Vanesa Jurasova, Katerina Veverova, Francesco Angelucci, Vaclav Matoska, Jakub Hort

**Affiliations:** 1 Memory Clinic, Department of Neurology, Second Faculty of Medicine, Charles University and Motol University Hospital, Prague, Czech Republic; 2 Edson College of Nursing and Health Innovation, Arizona State University, Phoenix, AZ, USA; 3 Department of Clinical Biochemistry, Hematology and Immunology, Homolka Hospital, Prague, Czech Republic

**Keywords:** Alzheimer's disease, *APOE*, biomarkers, α-Klotho, *KLOTHO*-VS heterozygosity

## Abstract

**Background:**

*KLOTHO*-VS heterozygosity (*KL*-VSHET) and soluble α-Klotho (sαKl) protein interfere with Alzheimer's disease (AD) pathophysiology, but the specific relationships remain unclear. This study explored these associations across the AD continuum, focusing on core AD biomarkers and markers of neurodegeneration, neuroinflammation, and synaptic dysfunction.

**Objective:**

We investigated whether 1) *KL*-VSHET is associated with lower AD biomarker burden (Aβ_42_, Aβ_42/40_ ratio, P-tau181, T-tau) and neurodegeneration (NfL); 2) sαKl relates to AD biomarkers, neurodegeneration (NfL), neuroinflammation (GFAP), and synaptic dysfunction (Ng); 3) associations vary by *APOE* ε4 status and clinical subgroup.

**Methods:**

Participants (n = 223) were categorized as cognitively healthy (n = 38), aMCI-AD (n = 94), and AD dementia (n = 91). *KLOTHO* genotyping was available for 128 participants; 138 had cerebrospinal fluid (CSF) and serum sαKl measurements; and 42 had both. Multiple linear regression evaluated associations between *KL*-VSHET, sαKl levels, and biomarkers, stratified by *APOE* ε4 status and clinical subgroup.

**Results:**

Overall, the associations between *KL*-VSHET and higher CSF Aβ_42_ and Aβ_42/40_ ratio were non-significant (*p*s ≥ 0.059) except when restricted to *APOE* ε4 carriers only (β = 0.11, *p *= 0.008 and β = 0.16, *p *= 0.033, respectively). Within clinical subgroups, *KL*-VSHET was positively associated with Aβ_42/40_ ratio only in aMCI-AD (β = 0.23, *p *= 0.034). No significant associations were observed between *KL*-VSHET and tau biomarkers or NfL. For sαKl, associations with biomarkers were non-significant except for a negative association of serum sαKl with P-tau181 in aMCI-AD (β = −0.25, p = 0.036) and a positive association with Aβ_42/40_ ratio in *APOE* ε4 non-carriers (β = 0.24 p = 0.047).

**Conclusions:**

*KL*-VSHET may help protect against amyloid pathology, particularly in the presence of *APOE* ε4, and regardless of *APOE* status in aMCI-AD.

## Introduction

Alzheimer's disease (AD) remains a significant challenge in the field of neuroscience due to its intricate pathophysiology and the complex interplay of various genetic, lifestyle, and biochemical factors. Among the genetic factors, the apolipoprotein allele 4 (*APOE* ε4) is recognized as the strongest genetic risk factor for sporadic AD.^1^ However, certain genetic variants may offer protective effects against AD pathology.

Recent research suggests that heterozygosity for the *KLOTHO*-VS (*KL*-VS) haplotype may be associated with a reduced burden of key AD biomarkers, such as cerebrospinal fluid (CSF) amyloid-β (Aβ) 42, total tau (T-tau), and phosphorylated tau (P-tau). This positive association has been observed in both cognitively normal individuals and those diagnosed with AD.^2–6^ Specifically, *KL*-VS heterozygosity (*KL*-VSHET) appears to mitigate age- and *APOE* ε4-related accumulation of Aβ_42_, T-tau, and P-tau, suggesting a potential protective role against AD pathology in *APOE* ε4 carriers.^6–12^ In addition, recent findings indicate that *KL*-VSHET may also be associated with reduced age-related neuroinflammatory and neurodegenerative profiles in cognitively unimpaired adults at risk for AD.^13^

*KL*-VSHET has further been linked to higher levels of soluble CSF and/or serum based soluble α-Klotho (sαKl) protein.^14–16^ Predominantly expressed in the kidneys and choroid plexus in the brain, α-Klotho exists in membrane-bound, secreted and soluble forms.^17,18^ This protein has garnered attention for its neuroprotective properties, which include mitigation of oxidative stress, reducing neuroinflammation, modulation of signaling pathways and enhancement of synaptic plasticity.^19,20^ Among its mechanisms, α-Klotho enhances the expression of antioxidant enzymes,^20^ while also decreasing pro-inflammatory markers, thereby reducing inflammation triggered by Aβ exposure.^19^ This antioxidative and anti-inflammatory action is particularly relevant in the context of AD, where oxidative damage and chronic inflammation are hallmarks of the disease Additionally, α-Klotho enhances synaptic plasticity and cognitive function by modulating N-methyl-D-aspartate receptors, critical for long-term potentiation.^16,21,22^ Its overexpression in the hippocampus improves synaptic function and cognitive performance in animal models,^21,22^ suggesting its role in promoting cognitive resilience alongside neuroprotection.

Cognitively unimpaired older adults show higher CSF sαKl levels compared to those with AD.^4^ However, prior research investigating the relationship between sαKl levels (serum/plasma and CSF) and AD biomarkers across the AD continuum has yielded mixed results. Driscoll and colleagues found no association between serum sαKl and AD biomarkers (CSF Aβ_42/40_, Aβ_42_, T-tau, P-tau) in pre-symptomatic individuals.^23^ Conversely, Ren and colleagues reported a negative correlation between plasma sαKl and CSF Aβ_42_, but not T-tau or P-tau181.^24^ Grøndvedt and colleagues presented a more complex picture: CSF sαKl positively correlated with CSF Aβ_42_ and negatively with CSF T-tau and P-tau181, supporting a potential modulatory role in AD pathology. Plasma sαKl, however, only linked negatively to CSF T-tau and P-tau181, highlighting potential differences between serum and CSF sαKl dynamics.^4^ These discrepancies underscore the need for further research to elucidate sαKl's role in AD and understand the potential distinctions between serum and CSF sαKl dynamics.

In light of these findings, our study aims to build on previous work by examining the relationship between *KL*-VSHET, CSF and serum sαKl levels, core AD markers and additional biomarkers of non-specific processes involved in AD pathophysiology, including glial fibrillary acidic protein (GFAP; for neuroinflammation), neurofilament light chain (NfL; for neurodegeneration), and neurogranin (Ng; for synaptic dysfunction). By incorporating these non-specific biomarkers alongside the core AD markers, this study offers a novel approach to understanding the interaction between *KL*-VSHET, sαKl, and AD-related pathology. Specifically, we set out to test the following hypotheses in a patient sample from the Czech Brain Aging Study:

1. *KL*-VSHET is associated with reduced burden of AD pathology (as measured by CSF Aβ_42_, Aβ_42/40_ ratio, T-tau and P-tau181) and neurodegeneration (as measured by NfL);
2. Higher CSF sαKl protein levels are independently associated with lower AD pathology burden and improved profiles for biomarkers of non-specific processes, regardless of *KL*-VS haplotype.

We hypothesized that the protective association of *KL*-VSHET and higher sαKl protein levels would be present only in individuals who are also carriers of the *APOE* ε4 risk variant. By addressing these hypotheses, our study aims to contribute to the understanding of genetic and biochemical factors that may mitigate the risk of AD, potentially guiding future therapeutic strategies.

## Methods

### Participants

In total, 223 participants from the Czech Brain Aging Study, a prospective multicenter study focusing on the neuroepidemiological characteristics of brain aging in the Czech Republic,^25^ met inclusion criteria for this study. All participants underwent standard neurological and laboratory evaluations within three months from the initial visit. All participants involved in this study signed written informed consent approved by the Motol University Hospital ethics committee.

Sample 1 (participants with available genotyping) included 128 participants. Of them, 55 met the clinical criteria for AD dementia,^26^ 55 met the clinical criteria for aMCI due to AD (aMCI-AD),^27^ and 18 were cognitively healthy participants. Demographic data are shown in Table 1.

**Table 1.** Characteristics of study participants in Sample 1 (participants with available genotyping).

	AD dementia patients (*n *= 55)	aMCI-AD patients (*n *= 55)	Controls (*n *= 18)	*P* AD dementia patients versus controls	*P* aMCI-AD patients versus controls
**Demographic characteristics**					
Female/Male	34/21	27/28	10/8	0.637	0.634
Age, y	70.8 ± 9.2	72.8 ± 8.5	66.6 ± 10.3	0.100	**0**.**012**
Education, y**	14.8 ± 2.9	15.0 ± 3.0	16.4 ± 2.8	**0**.**049**	0.086
*APOE* ε4 positive, n (%) *	36 (65.5)	29 (52.7)	8 (44.4)	0.114	0.634
KLVS-HET+, n (%)	15 (27.3)	9 (16.4)	8 (44.4)	0.173	**0**.**014**
MMSE score ***	19.8 ± 4.2	25.4 ± 2.4	28.6 ± 1.4	<0.001	<0.001
**AD biomarkers**					
CSF Aβ_42/40_	0.06 ± 0.05	0.08 ± 0.07	0.11 ± 0.05	**<0**.**001**	**<0**.**001**
CSF Aβ_42_, pg/mL	474.5 ± 158.2	500.3 ± 177.0	1080.6 ± 238.2	**<0**.**001**	**<0**.**001**
CSF Aβ_40_, pg/mL	8802.8 ± 3517.6	8742.0 ± 3840.1	9568.9 ± 3623.9	0.447	0.421
CSF T-tau, pg/mL	626.7 ± 361.4	637.8 ± 508.8	259.7 ± 118.4	**<0**.**001**	**<0**.**001**
CSF P-tau181, pg/mL	122.9 ± 80.3	116.7 ± 92.0	40.1 ± 23.0	**<0**.**001**	**<0**.**001**
CSF NfL, pg/mL ^+^	1641.7 ± 1152.7	1229.6 ± 515.5	968.9 ± 679.4	**0**.**021**	0.223
Serum NfL, pg/mL ^++^	30.7 ± 13.4	26.8 ± 14.6	17.1 ± 7.1	**<0**.**001**	**0**.**002**

Values are presented as mean ± SD except for gender and *APOE*. p values are comparisons using t-test for continuous variables and chi square test for categorical variables.

Aβ_42_: amyloid-β 42; Aβ_42/40_: amyloid-beta 42 to 40 ratio; AD: Alzheimer's disease; *APOE*: apolipoprotein E; CSF: cerebrospinal fluid; aMCI: amnestic mild cognitive impairment; MMSE: Mini-Mental State Examination; NfL: neurofilament light chain; P-tau181: phosphorylated tau 181; T-tau: total tau

* missing genotype data in *n *= 3 (1 aMCI-AD, 2 controls) ** missing education data in *n *= 6 (5 AD-dementia, 1 aMCI-AD) *** missing MMSE data in *n *= 17 (14 AD-dementia, 3 aMCI-AD)

+ missing CSF NfL data in *n *= 46 (24 AD-dementia, 17 aMCI-AD, 5 controls), ^++^ missing serum NfL data in *n *= 29 (13 AD-dementia, 14 aMCI-AD, 2 controls)

Sample 2 (participants with available CSF or serum-based sαKl protein) included 138 participants. Of them, 56 met the clinical criteria for AD dementia,^26^ 56 met the clinical criteria for aMCI-AD,^27^ and 26 were cognitively healthy participants. Demographic data are shown in Table 2.

**Table 2.** Characteristics of study participants in Sample 2 (participants with available CSF and serum-based sαKl protein).

	AD dementia patients (*n *= 56)	aMCI-AD patients (*n *= 56)	Controls (*n *= 26)	*P* AD dementia patients versus controls	*P* aMCI-AD patients versus controls
**Demographic characteristics**					
Female/Male	36/20	34/22	21/5	0.131	0.072
Age, y	74.8 ± 5.5	73.4 ± 4.9	60.7 ± 8.1	**<0**.**001**	**<0**.**001**
Education, y**	13.9 ± 2.9	14.5 ± 3.2	15.3 ± 2.7	**0**.**040**	0.287
*APOE* ε4 positive, n (%) *	35 (62.5)	43 (76.8)	6 (23.1)	**<0**.**001**	**<0**.**001**
MMSE score ***	18.7 ± 4.6	25.0 ± 2.7	29.0 ± 0.9	**<0**.**001**	**<0**.**001**
**Biomarkers**					
sαKl					
CSF sαKl, pg/ml	1196.3 ± 262.4	1272.6 ± 227.5	1338.9 ± 210.52	**0**.**018**	0.225
Serum sαKl, pg/ml	970.1 ± 321.6	1003.6 ± 397.2	1165.6 ± 475.2	0.081	0.161
AD biomarkers					
CSF Aβ_42/40_	0.05 ± 0.02	0.04 ± 0.02	0.14 ± 0.06	**<0**.**001**	**<0**.**001**
CSF Aβ_42_, pg/mL	443.6 ± 232.2	435.2 ± 219.3	1141.4 ± 474.8	**<0**.**001**	**<0**.**001**
CSF Aβ_40_, pg/mL	9818.0 ± 3341.2	10769.5 ± 3846.0	9747.3 ± 4165.0	0.942	0.311
CSF T-tau, pg/mL	699.6 ± 438.7	602.4 ± 276.1	215.5 ± 107.4	**<0**.**001**	**<0**.**001**
CSF P-tau181, pg/mL	110.5 ± 69.8	108.4 ± 70.2	32.0 ± 13.1	**<0**.**001**	**<0**.**001**
CSF NfL, pg/mL ^+^	1763.8 ± 974.1	1331.5 ± 921.7	649.3 ± 531.8	**<0**.**001**	**<0**.**001**
CSF Ng, pg/mL ^++^	275.0 ± 109.0	263.0 ± 103.9	175.1 ± 73.8	**0**.**002**	**0**.**004**
Serum NfL, pg/mL ^+++^	41.8 ± 24.6	39.1 ± 33.2	15,0 ± 9.9	**<0**.**001**	**<0**.**001**
Serum GFAP, pg/mL ^++++^	511.1 ± 280.9	528.9 ± 233.4	155.9 ± 50.9	**<0**.**001**	**<0**.**001**

Values are presented as mean ± SD except for gender and *APOE*. p values are comparisons using t-test for continuous variables and chi square test for categorical variables.

Aβ_42_: amyloid-beta 42; Aβ_42/40_: amyloid-beta 42 to 40 ratio; AD: Alzheimer's disease; *APOE*: apolipoprotein E; CSF: cerebrospinal fluid; aMCI: amnestic mild cognitive impairment; GFAP: glial fibrillary acidic protein; MMSE: Mini-Mental State Examination; NfL: neurofilament light chain; Ng: neurogranin; P-tau181: phosphorylated tau 181; T-tau: total tau.

*missing genotype data in *n *= 16 (5 AD-dementia, 1 aMCI-AD, 10 controls) ** missing education data in n = 5 (5 AD-dementia) *** missing MMSE data in *n *= 3 (3 AD-dementia).

+ missing CSF NfL data in *n *= 11 (8 AD-dementia, 1 aMCI-AD, 2 controls), ^++^ missing CSF Ng data in *n *= 44 (15 AD-dementia, 14 aMCI-AD, 15 controls), ^+++^ missing serum NfL data in *n *= 5 (1 AD-dementia, 1 aMCI-AD, 3 controls), ^++++^ missing serum GFAP data in *n *= 68 (24 AD-dementia, 34 aMCI-AD, 10 controls).

There was an overlap of 42 participants who had both *KLOTHO* genotyping and CSF/serum-based sαKl measurements. This overlap consisted of 20 participants with AD dementia, 16 participants with aMCI-AD, and 6 cognitively healthy participants.

All participants included in the study underwent lumbar puncture to determine the status of AD biomarkers. Participants diagnosed with AD dementia or aMCI-AD had a positive AD biomarker status based on CSF (reduced Aβ_42_ and elevated P-tau181 [<620 pg/mL and >61 pg/mL, respectively]). Cognitively unimpaired controls had normal levels of AD biomarkers in CSF.

Participants diagnosed with AD dementia met the NIA-AA 2011 criteria for dementia due to AD,^26^ based on progressive decline in at least two cognitive domains (i.e., ≥ 1.5 standard deviations (SD) lower memory test score than the age- and education-adjusted norms as well as similarly low score in at least one non-memory cognitive test), alongside evidence of AD pathophysiology, and significant impairment in the ability to perform daily activities.

Participants diagnosed with aMCI-AD met the NIA-AA 2011 criteria for MCI^27^ based on subjective reports of memory decline compared to their prior level, objective evidence of memory impairment (i.e., ≥ 1.5 SDs lower score than the age- and education-adjusted norms in any memory test), preserved independence in daily activities, and the absence of dementia.

Cognitively unimpaired controls (n = 38) were recruited from:

**a)** Patients from the Department of Neurology who underwent a lumbar puncture to rule out central nervous system inflammation (n = 28). These individuals had normal CSF analysis, no signs of systemic inflammation, and no evidence of hippocampal atrophy on magnetic resonance imaging (MRI). Additionally, they displayed normal cognitive function (scores > 1.5 SD above age- and education-adjusted norms on all cognitive tests) and reported no subjective cognitive complaints. CSF biomarkers for AD were also within normal ranges.
**b)** Czech Brain Aging Study patients followed for subjective cognitive decline (n = 10). While reporting cognitive difficulties that motivated them to seek medical help, they exhibited unimpaired activities of daily living and normal cognitive function (>1.5 SD above age- and education-adjusted norms).^28^ They had no evidence of hippocampal atrophy on MRI and had normal AD biomarkers in CSF or PET scans.

### Exclusion criteria

The study excluded participants with pre-existing neurological or psychiatric conditions, including depression (≥6 points on the 15-item Geriatric Depression Scale),^29^ and patients with severe white matter vascular lesions on MRI (Fazekas score > 2 points).^30^ Participants suffering from renal failure were excluded since kidneys are a major site of α-Klotho production. Participants carrying the ε2/ε4 (n = 6) or ε2/ε3 (n = 5) allele combination were also excluded since the ε2 allele has been shown to be protective against AD. Four participants were excluded from the study sample because of *KL*-VS homozygosity.

### Genotyping

*APOE* and *KLOTHO* genotypes were determined at the Department of Clinical Biochemistry, Hematology and Immunology, Homolka Hospital, Prague, Czech Republic.

DNA isolation was performed by Zybio Nucleic extraction kit WB-B from whole blood samples according to manufacturer's protocol (Zybio, Chongqing, China).

*APOE* genotyping was performed according to IdahoTech protocol (Luna Probes Genotyping Apolipoprotein [ApoE] Multiplexed Assay) for high-resolution melting analysis (HRM).^31,32^ The *APOE* ε4 carrier group included *APOE* ε4 homozygotes (ε4/ε4) and heterozygotes (ε3/ε4).

The analysis of *KL*-VS haplotype was done also by HRM analysis of single nucleotide polymorphism rs9536314 (G/T). The reaction was performed with LightScanner Master mix (BioFire Diagnostics, SLC., USA) according to manufacturer's PCR reaction conditions with forward and reverse primers: KL1F 5´- ATAACCTTTCATCTATTCTGC-3´; KL1R 5´- AAGTCAGCAGTTCCTTTG-3´; Temperature profile was: 95°C for 2 min followed by 40 cycles of 95°C/30 s; 63°C/30 s; 72°C/30 s; Melting 60°C–90°C. HRM analysis was performed on LightScanner (IdahoTech).

### CSF and blood collection and processing

Blood samples were drawn by venipuncture, allowed to clot at room temperature for 15 min, and centrifuged at 1700 × g at 20°C for 5 min within 30 min of collection. CSF was obtained by lumbar puncture in 8-mL polypropylene tubes, gently mixed, and centrifuged at 1700 × g at 20°C for 5 min within 30 min of collection. CSF and serum supernatant were aliquoted in polypropylene tubes of 0.5 ml and stored at −80°C. Before analysis, serum and CSF samples stored at −80°C were thawed at room temperature and vortexed for 15 s.

### Immunological assays

CSF levels of Aβ_42_, Aβ_40_, T-tau, P-tau181, and Ng were measured using a LUMIPULSE^®^ G600II instrument (Fujirebio, Ghent, Belgium).^33^ CSF Aβ_42/40_ ratio was calculated and used as an additional dependent variable. Commercial enzyme-linked immunosorbent assay (ELISA) was used to measure levels of NfL in CSF and serum (UmanDiagnostics, 10-7001CE and 20-8002RUO). Serum GFAP concentration was measured using the Quanterix Simoa Neurology 4-Plex B following the manufacturer's protocol (Quanterix, Billerica, MA).

Protein levels of sαKl in serum and CSF were quantified using commercially available enzyme-linked immunosorbent assay (ELISA) kit (Immuno-Biological Laboratories Co Ltd, Japan; cat. no. JP27998) following the manufacturer's instructions. Serum and CSF samples were measured in duplicate and were analyzed undiluted. At the end of the assay, absorbances were read at 450 nm using a microplate reader (Dynex Technologies, Virginia, USA), and the protein concentration was calculated by comparison with a standard curve. The intra-assay coefficient of variance (CV%) was <3% and the inter-assay CV was <8%.

### Statistical analyses

We used the independent samples t-test to evaluate between-group differences in age, years of education, and global cognitive functioning as assessed by Mini-Mental State Examination (MMSE), and the χ2 test to evaluate differences in frequencies for sex, *APOE* ε4, and *KL*-VSHET. Independent samples t-tests were used to compare mean sαKl levels between *KL*-VSHET and wild-type individuals and to compare MMSE and biomarker levels between *APOE* ε4 carriers and non-carriers.

Normality was assessed through the inspection of histograms, skewness, and the Shapiro-Wilks test of normality. Serum sαKl, CSF core AD biomarkers (CSF Aβ_42_, Aβ_40_, T-tau, P-tau181) and biomarkers of non-specific processes involved in AD pathophysiology (GFAP, Ng, NfL) had nonnormal distribution; therefore, log transformation was applied.

To assess the association between *KL*-VSHET, core AD biomarkers (Aβ_42_, Aβ_40_, T-tau, P-tau181) and biomarker of neurodegeneration (NfL), we used multiple linear regression models with each biomarker as the dependent variable and *KL*-VS haplotype as the independent variable. Age and sex were included as covariates in all models.

Similarly, we examined the relationship between CSF and serum sαKl protein levels, core AD biomarkers (Aβ_42_, Aβ_40_, T-tau, P-tau181) and biomarkers of non-specific processes involved in AD pathophysiology (GFAP, Ng, NfL) using separate multiple linear regression models for each biomarker. Each model included the respective AD biomarker as the dependent variable, CSF or serum sαKl level as the independent variable, and age and sex as covariates.

## Results

### Sample characteristics

Group demographic and clinical characteristics of Sample 1 (participants with available genotyping) are presented in Table 1. There was no significant difference in sex distribution among the study groups. Controls were significantly younger compared to aMCI-AD patients (*p *= 0.012). Participants diagnosed with AD dementia had fewer years of education than the controls (*p *= 0.049). The proportion of *KL*-VSHET individuals was significantly lower in the aMCI-AD group compared to the control group (*p *= 0.014), but not in the AD dementia group compared to the control group (*p *= 0.173). Finally, individuals with aMCI-AD and AD dementia had significantly lower MMSE score compared to controls (both *p*s < 0.001).

Group demographic and clinical characteristics of Sample 2 (participants with available CSF or serum based sαKl protein) are presented in Table 2. Both the aMCI-AD and AD dementia groups were significantly older than the controls (both *p*s < 0.001). The AD dementia group had significantly fewer years of education than the controls (*p *= 0.040). The percentage of *APOE* ε4 positive individuals was significantly higher in the AD dementia and aMCI-AD groups compared to controls (both *p*s < 0.001). Finally, MMSE score was significantly lower in the aMCI-AD and AD dementia groups compared to controls (both *p*s < 0.001).

### Group differences by APOE ε4 status in Sample 1

**Figure 1.**
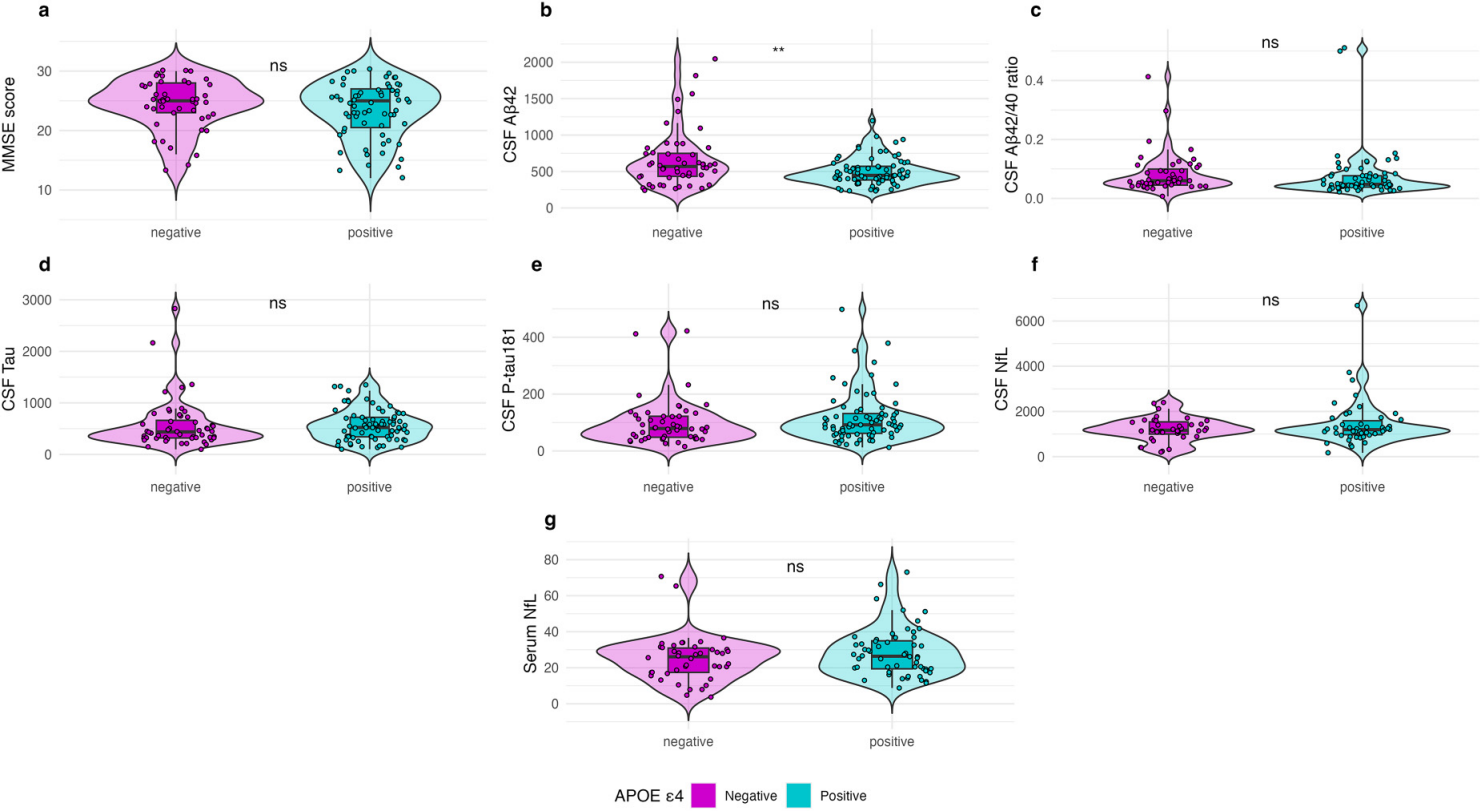
Group Differences by *APOE* ε4 Status in Sample 1 (participants with available genotyping).

MMSE scores did not differ between the *APOE* ε4 non-carriers and *APOE* ε4 carriers (*p *= 0.269). CSF Aβ_42_ levels were significantly higher in the *APOE* non-carriers compared to the *APOE* ε4 carriers (*p *= 0.016). However, no significant differences between the groups were found for the CSF Aβ_42/40_ ratio (*p *= 0.203), CSF T-tau (*p *= 0.738), or CSF P-tau181 (*p *= 0.801), CSF NfL (*p *= 0.183) or serum NfL levels (*p *= 0.202) (Figure 1).

### Group differences by APOE ε4 status in Sample 2

**Figure 2.**
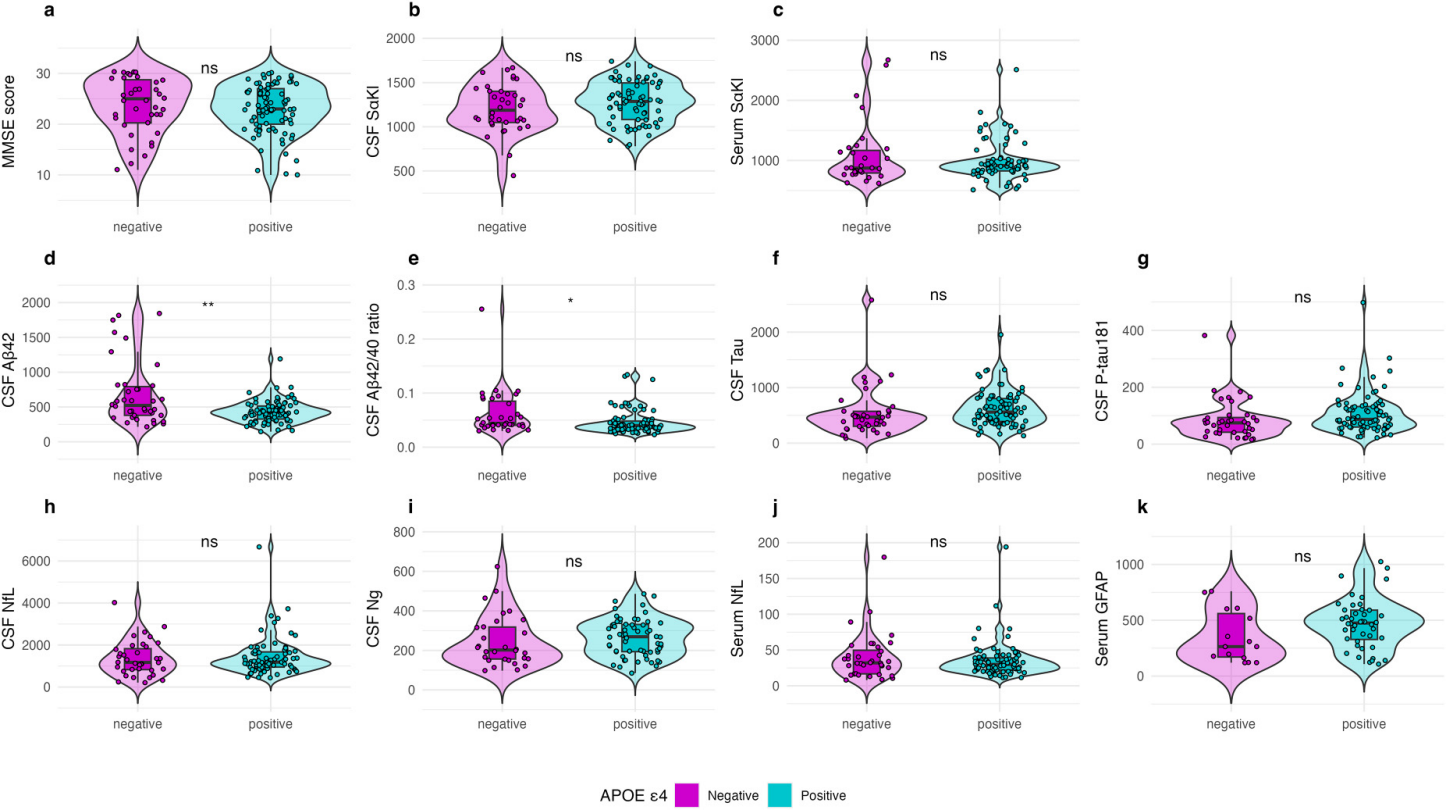
Group Differences by *APOE* ε4 Status in Sample 2 (participants with available CSF and serum-based sαKl protein).

No significant differences were observed in MMSE scores between the *APOE* ε4 non-carriers and *APOE* ε4 carriers (*p *= 0.532). Similarly, sαKl levels in both CSF and serum did not differ significantly between the *APOE* groups (*p *= 0.183 and *p *= 0.516, respectively). Additionally, no significant group differences were observed in CSF P-tau 181 (*p *= 0.091), CSF NfL (*p *= 0.341), CSF Ng (*p *= 0.237), or serum NfL (*p *= 0.776). However, significant group differences were found for CSF Aβ_42_ and the Aβ_42/40_ ratio, which were higher in the *APOE* ε4 non-carriers compared to the *APOE* ε4 carriers (*p *= 0.002, *p *= 0.007, respectively). CSF T-tau and serum GFAP levels showed a trend toward being higher in the *APOE* ε4 carriers, although this difference did not reach statistical significance (*p *= 0.064 and *p *= 0.083, respectively) (Figure 2).

### Association of *KL*-VSHET with core AD biomarkers and NfL

Controlling for age and sex, *KL*-VSHET showed a non-significant trend towards a positive association with Aβ_42/40_ ratio when using the entire sample (β = 0.10, *p *= 0.091). Stratifying by *APOE* ε4 revealed this association was significant in *APOE* ε4 carriers (β = 0.16, *p *= 0.033), which included both ε4/ε4 homozygotes and ε3/ε4 heterozygotes, but not in *APOE* ε4 non-carriers (β = 0.03, *p *= 0.786). When analyzing by clinical subgroups, *KL*-VSHET was significantly associated with the Aβ_42/40_ ratio, but only in the aMCI-AD group (β = 0.23, p = 0.034).

A similar pattern emerged for Aβ_42_, with a non-significant trend towards positive association between carrying the *KL*-VSHET haplotype and higher Aβ_42_ levels in the entire sample (β = 0.08, *p *= 0.059), a significant association in *APOE* ε4 carriers (β = 0.11, *p *= 0.008), and a non-significant association in *APOE* ε4 non-carriers (β = 0.03, *p *= 0.683).

No significant associations were detected for *KL*-VSHET with T-tau, P-tau181 or NfL levels in the entire sample or in the *APOE* ε4 or clinical subgroups (all *p*s > 0.05). The regression coefficients of each of the linear regression models showing *KL*-VSHET in relation to the levels of biomarkers are presented in Table 3.

**Table 3.** Linear regression models showing the association of KL-VS heterozygosity with levels of biomarkers in the entire sample and by APOE ε4 and clinical subgroups.

	Entire sample				*APOE* ε4 carriers				*APOE* ε4 non-carriers		
Biomarker	β	SE	*p*		β	SE	*p*		β	SE	*p*
CSF Aβ_42/40_ ratio	0.02	0.02	0.091		0.04	0.02	**0**.**033**		0.01	0.03	0.786
CSF Aβ_42_	101.2	63.2	0.059		148.61	48.5	**0**.**008**		50.1	143.8	0.683
CSF T-tau	−131.6	87.8	0.151		−103.33	78.0	0.212		−28.0	169.1	0.995
CSF P-tau181	−33.9	51.1	0.404		21.59	22.9	0.992		−112.7	134.9	0.508
CSF NfL	−143.8	229.4	0.880		−246.52	374.9	0.766		18.7	210.0	0.493
Serum NfL	−4.5	3.2	0.145		−3.75	4.2	0.185		−6.1	5.3	0.407

**Table:** 

	AD dementia				aMCI due to AD				Controls		
Biomarker	β	SE	*p*		β	SE	*p*		β	SE	*p*
CSF Aβ_42/40_ ratio	−0.01	0.02	0.708		0.07	0.04	**0**.**034**		−0.02	0.03	0.649
CSF Aβ_42_	54.95	49.83	0.340		125.62	62.86	0.089		−301.02	223.03	0.159
CSF T-tau	−121.01	103.23	0.156		−72.71	195.58	0.901		60.01	62.29	0.475
CSF P-tau181	7.21	23.00	0.922		−44.82	138.24	0.967		12.87	11.87	0.443
CSF NfL	−224.40	556.97	0.820		−43.31	242.35	0.908		423.06	418.98	0.173
Serum NfL	−3.03	5.24	0.260		−5.34	6.36	0.662		−1.24	4.08	0.321

The *p*-values are derived from analyses using log-transformed biomarker values to handle non-normality. The coefficients (β) and standard errors (SE) represent the results from analyses using non-transformed biomarker values for easier interpretation.

Aβ_42_: amyloid-beta 42; Aβ_42/40_: amyloid-beta 42 to 40 ratio; AD: Alzheimer's disease; *APOE*: apolipoprotein E; CSF: cerebrospinal fluid; *KL*-VS: *KLOTHO*-VS; NfL: neurofilament light chain; P-tau181: phosphorylated tau 181; SE: standard error of measurement; T-tau: total tau.

### Association of sαKl protein levels with core AD biomarkers and biomarkers of non-specific processes involved in AD pathophysiology

No significant associations were identified between CSF sαKl levels and any of the core AD biomarkers or biomarkers of neurodegeneration (NfL), neuroinflammation (GFAP) and synaptic dysfunction (Ng) in either the entire sample or within the *APOE* ε4 or clinical subgroups (Table 4).

**Table 4.** Linear regression models showing the association of **CSF sαKl** with levels of core AD and non-specific biomarkers involved in AD pathophysiology in the entire sample and by APOE ε4 and clinical subgroups.

	Entire sample				*APOE* ε4 carriers				*APOE* ε4 non-carriers		
Biomarker	β	SE	p		β	SE	p		β	SE	p
CSF Aβ_42/40_ ratio	−0.14	0.09	0.096		−0.02	0.07	0.767		−0.12	0.14	0.399
CSF Aβ_42_	−0.14	0.09	0.109		0.01	0.08	0.955		−0.25	0.19	0.197
CSF T-tau	0.06	0.09	0.494		0.02	0.10	0.856		0.12	0.16	0.438
CSF P-tau181	0.04	0.09	0.643		−0.01	0.11	0.930		0.09	0.16	0.573
CSF NfL	0.03	0.08	0.696		−0.06	0.10	0.479		0.06	0.17	0.719
CSF Ng	−0.03	0.12	0.778		−0.01	0.14	0.998		−0.09	0.24	0.713
Serum NfL	−0.08	0.08	0.300		−0.13	0.09	0.138		0.01	0.17	0.972
Serum GFAP	0.02	0.10	0.847		−0.07	0.12	0.550		0.69	0.37	0.092

**Table:** 

	AD dementia				aMCI due to AD				Controls		
Biomarker	β	SE	p		β	SE	p		β	SE	p
CSF Aβ_42/40_ ratio	−0.05	0.10	0.661		−0.10	0.08	0.474		−0.35	0.23	0.153
CSF Aβ_42_	−0.08	0.13	0.515		−0.14	0.10	0.160		−0.14	0.22	0.525
CSF T-tau	0.10	0.13	0.433		0.03	0.11	0.774		0.10	0.24	0.694
CSF P-tau181	0.09	0.14	0.527		0.03	0.11	0.795		−0.05	0.14	0.704
CSF NfL	0.02	0.11	0.880		0.05	0.11	0.644		0.02	0.19	0.900
CSF Ng	0.14	0.19	0.457		−0.11	0.18	0.551		0.13	0.57	0.830
Serum NfL	−0.10	0.11	0.377		−0.03	0.13	0.790		0.01	0.22	0.975
Serum GFAP	0.22	0.21	0.316		0.12	0.13	0.384		0.05	0.15	0.731

Aβ_42_: amyloid-beta 42; Aβ_42/40_: amyloid-beta 42 to 40 ratio; AD: Alzheimer's disease; *APOE*: apolipoprotein E; CSF: cerebrospinal fluid; GFAP: glial fibrillary acidic protein; NfL: neurofilament light chain; Ng: neurogranin; P-tau181: phosphorylated tau 181; sαKl: soluble α-Klotho protein; SE: standard error of measurement; T-tau: total tau.

Similarly, serum sαKl levels did not show a significant association with any of the core AD biomarkers or biomarkers of non-specific processes involved in AD pathophysiology when analyzing the entire sample. When stratifying the analysis based on *APOE* ε4 carriership, serum sαKl levels were positively associated with Aβ_42/40_ ratio, but only in *APOE* ε4 non-carriers (β = 0.24, *p *= 0.047) (Table 5). When stratifying the analysis based on clinical subgroups, serum sαKl levels were negatively related to P-tau181 in the aMCI-AD group (β = −0.25, p = 0.036). No significant associations were observed for any of the other biomarkers in either *APOE* ε4 or clinical subgroups.

**Table 5.** Linear regression models showing the association of **serum sαKl** with levels of core AD and non-specific biomarkers involved in AD pathophysiology in the entire sample and by APOE ε4 and clinical subgroups.

	Entire sample				*APOE* ε4 carriers				*APOE* ε4 non-carriers		
Biomarker	β	SE	p		β	SE	p		β	SE	p
CSF Aβ_42/40_ ratio	0.12	0.09	0.190		0.09	0.09	0.338		0.24	0.12	**0**.**047**
CSF Aβ_42_	0.09	0.09	0.351		0.05	0.09	0.616		0.10	0.17	0.559
CSF T-tau	−0.01	0.09	0.951		−0.03	0.09	0.712		−0.11	0.16	0.470
CSF P-tau181	−0.09	0.09	0.260		−0.12	0.10	0.229		−0.14	0.17	0.422
CSF NfL	−0.01	0.09	0.880		0.04	0.11	0.687		−0.10	0.16	0.534
CSF Ng	−0.03	0.11	0.756		−0.03	0.13	0.793		−0.06	0.22	0.796
Serum NfL	−0.08	0.08	0.341		0.01	0.10	0.964		−0.09	0.13	0.470
Serum GFAP	−0.01	0.11	0.879		0.03	0.13	0.850		0.09	0.21	0.692

**Table:** 

	AD dementia				aMCI due to AD				Controls		
Biomarker	β	SE	p		β	SE	p		β	SE	p
CSF Aβ_42/40_ ratio	0.16	0.11	0.148		0.05	0.10	0.624		0.13	0.18	0.477
CSF Aβ_42_	0.14	0.13	0.277		−0.12	0.12	0.291		0.24	0.15	0.113
CSF T-tau	0.13	0.12	0.286		−0.15	0.12	0.214		0.19	0.19	0.325
CSF P-tau181	0.03	0.13	0.800		−0.25	0.12	**0**.**036**		0.10	0.13	0.423
CSF NfL	−0.01	0.13	0.991		−0.07	0.12	0.535		0.18	0.15	0.232
CSF Ng	−0.01	0.16	0.980		−0.19	0.17	0.272		0.29	0.27	0.315
Serum NfL	0.06	0.13	0.647		−0.18	0.14	0.191		−0.01	0.15	0.953
Serum GFAP	0.01	0.14	0.972		0.09	0.15	0.560		0.06	0.10	0.561

Aβ_42_: amyloid-beta 42; Aβ_42/40_: amyloid-beta 42 to 40 ratio; AD: Alzheimer's disease; *APOE*: apolipoprotein E; CSF: cerebrospinal fluid; GFAP: glial fibrillary acidic protein; NfL: neurofilament light chain; Ng: neurogranin; P-tau181: phosphorylated tau 181; sαKl: soluble α-Klotho protein; SE: standard error of measurement; T-tau: total tau.

### Association of KL*-*VS haplotype with sαKl protein levels

**Figure 3.**
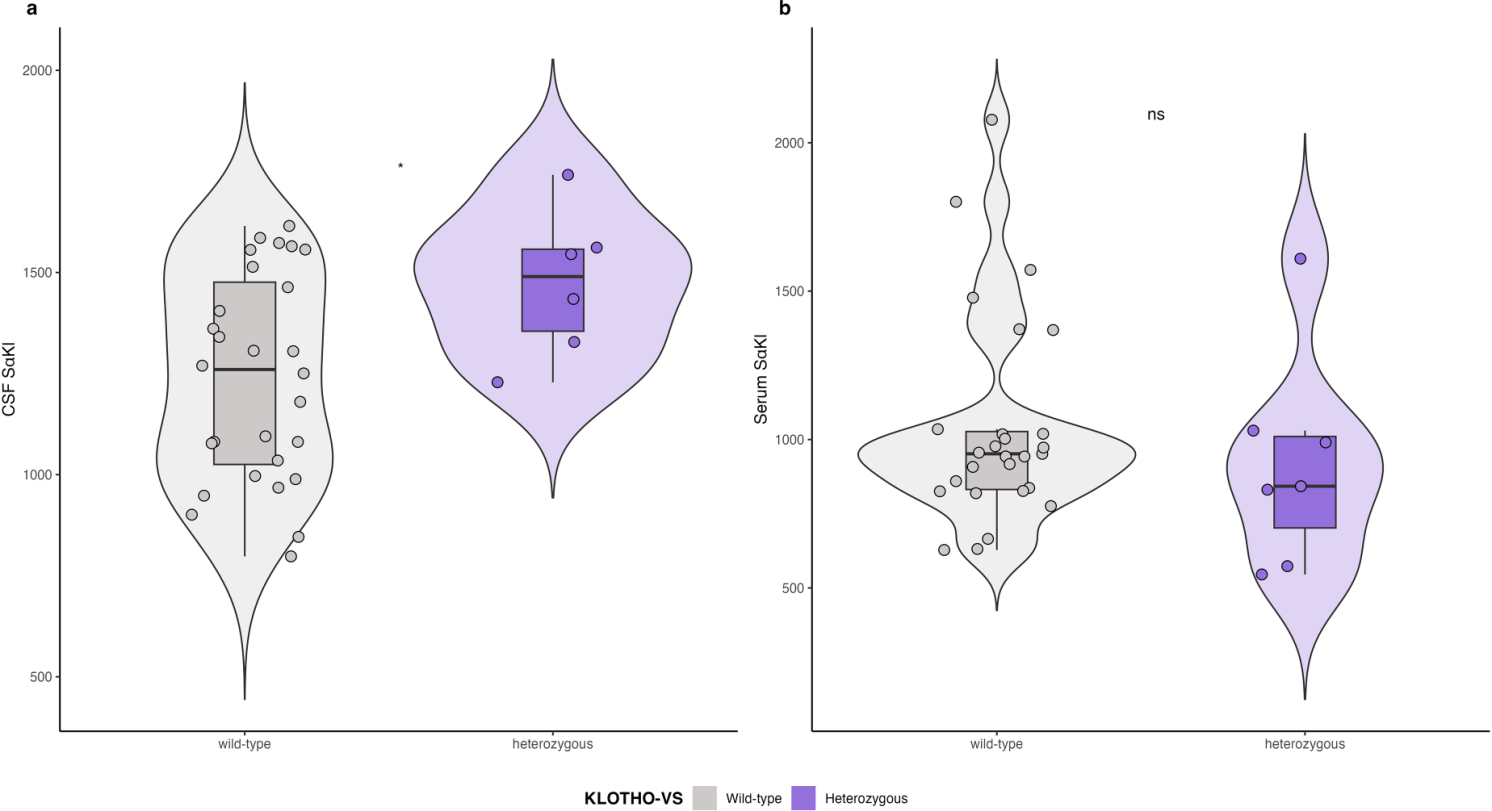
CSF and serum sαKl protein levels by *KL*-VS haplotype.

Additionally, we explored the relationship between *KL*-VS haplotype and sαKl levels in both CSF and serum. In the subset of participants with both *KL*-VS haplotyping and sαKl level measurements, we found a significant difference in CSF sαKl levels between *KL*-VSHET individuals and those with the wild-type genotype. Specifically, *KL*-VSHET individuals exhibited higher CSF sαKl levels compared to wild-type individuals (1473.2 ± 183.0 pg/mL versus 1237.9 ± 254.9 pg/mL, *p *= 0.025). In contrast, sαKl levels in serum showed no significant difference between the two groups (*p *= 0.37) (Figure 3).

## Discussion

This study aimed to investigate the protective role of *KL*-VSHET and sαKl protein levels on AD biomarker burden across the AD continuum, including both core AD biomarkers and biomarkers of non-specific processes like neuroinflammation, neurodegeneration and synaptic dysfunction. We found that *KL*-VSHET showed a non-significant trend towards a positive association with CSF Aβ_42/40_ ratio and Aβ_42_ levels in the whole sample. However, significant associations were only observed in *APOE* ε4 carriers, whereas the association was essentially non-existent among *APOE* ε4 non-carriers, suggesting that any potential protective effect of *KL*-VSHET on Aβ pathology may operate as a function of *APOE* ε4 status. These findings align with previous studies demonstrating that *KL*-VSHET is associated with lower Aβ burden specifically in cognitively normal individuals carrying the *APOE* ε4 risk allele.^5–10^ Notably, our results suggest that this protection may extend to individuals already diagnosed with clinically symptomatic AD, as we observed a significant positive association between KL-VSHET and Aβ_42/40_ ratio specifically in the aMCI due to AD group. This aligns with Cook and colleagues, who found *KL*-VSHET carriers exhibited consistent patterns of reduced Aβ burden across the AD continuum,^2^ suggesting a broader effect beyond cognitively unimpaired individuals.

Recent evidence highlights a regionally specific relationship between α-Klotho and apoE protein levels in the brain, particularly under environmental stress. Using irradiated Rhesus macaques, Kundu et al.^34^ demonstrated a strong positive correlation between α-Klotho and apoE protein levels in the amygdala, suggesting a potential adaptive response to stress. However, no such relationship was observed in the prefrontal cortex, and α-Klotho levels did not predict regional brain volumes or change with age in this sample. While the results warrant replication in human studies, these findings indicate that the interplay between α-Klotho and apoE may be region-specific and context-dependent. Although our study did not examine this relationship directly, the results underscore the importance of investigating potential synergistic effects of α-Klotho and apoE on brain structure and function, particularly in regions implicated in AD, such as the limbic system.

While we observed a positive association between *KL*-VSHET and Aβ biomarkers, we did not detect significant associations between *KL*-VSHET and tau biomarkers (T-tau or P-tau181) or NfL, a neurodegeneration marker, in our study sample. Driscoll et al.^13^ found that *KL*-VSHET attenuated age-related neurodegeneration as measured by CSF NfL levels in cognitively unimpaired individuals at risk for AD. However, the absence of an association with NfL in our sample, which included individuals across various stages of AD, may suggest that the neuroprotective effects of *KL*-VSHET on markers of neurodegeneration are more prominent at earlier, preclinical stages. This finding may indicate that *KL*-VSHET selectively modulates Aβ processing or clearance pathways, with its effects on neurodegeneration markers like NfL diminishing as the disease progresses or as AD pathology becomes more advanced.

Recent findings indicate that both Aβ and tau biomarkers in CSF reach plateau levels at different stages of the disease, reducing their utility in monitoring AD pathology as the disease progresses.^35^ In our sample, however, we observed significant differences in the levels of all CSF AD biomarkers between AD dementia and aMCI-AD. Specifically, both Aβ_42_ and Aβ_42/40_ ratio, as well as T-tau and P-tau181 levels, changed from aMCI to dementia. This suggests that while both types of biomarkers are dynamic over the disease course in our sample, their associations with *KL*-VSHET might differ due to underlying mechanisms. One possible explanation for this discrepancy is that *KL*-VSHET may specifically modulate pathways related to Aβ processing or clearance, rather than tau phosphorylation and aggregation. Similarly, Cook et al. observed a non-significant trend for reduced tau burden in cognitively normal *KL*-VSHET carriers but no effect in individuals with aMCI or dementia due to AD.^2^ Conversely, Neitzel and colleagues reported a protective effect of *KL*-VSHET against amyloid-dependent tau accumulation specifically in patients with aMCI-AD, particularly *APOE* ε4 carriers.^6^ Finally, some studies report no significant associations between *KL*-VSHET and both Aβ and tau biomarkers (T-tau, P-tau181) among AD patients.^4,5^ These discrepancies highlight the need for further research with larger and more diverse cohorts, focusing on longitudinal changes and stratifying by AD stage to definitively elucidate the complex role of *KL*-VSHET in both Aβ and tau pathology across the AD continuum.

Contrary to our hypothesis, we did not observe any significant associations between CSF sαKl and any of the core AD or non-specific biomarkers involved in AD pathophysiology, including NfL, GFAP, and Ng. Serum sαKl similarly exhibited no significant associations in the overall sample but showed a positive association with CSF Aβ_42/40_ ratio only in *APOE* ε4 non-carriers and a negative association with CSF P-tau181 only in aMCI due to AD patients. Our findings contrast with Grøndvedt et al., who reported a positive association of CSF sαKl with CSF Aβ_42_ and a negative association with CSF T-tau and P-tau, while plasma sαKl only correlated negatively with CSF T-tau and P-tau.^4^ Ren et al. observed a negative correlation between plasma Klotho and CSF Aβ_42_, but not any of the other investigated CSF biomarkers (T-tau, P-tau181, NfL) in a mixed sample of cognitively unimpaired individuals and AD patients,^24^ suggesting that higher sαKl levels are associated with greater Aβ accumulation and potentially more advanced AD pathology. Driscoll et al. reported no differences in serum sαKl between pre-symptomatic individuals who were positive vs. negative for Aβ or tau,^23^ suggesting no clear association between sαKl levels and early AD pathology. This aligns with our lack of significant findings in the entire sample. However, unlike the pre-symptomatic cohort studied by Driscoll and colleagues,^23^ our participants exhibited advanced AD pathology.

While no previous studies have examined the association between sαKl protein levels and biomarkers like NfL, GFAP, or Ng, Driscoll et al. investigated whether the *KL*-VSHET genotype influences these markers in cognitively unimpaired individuals at risk for AD.^13^ Their findings demonstrated that *KL*-VSHET attenuated the deleterious effects of age on CSF levels of Ng and GFAP, suggesting that *KL*-VSHET may play a protective role in synaptic function and neuroinflammation during early disease stages.^13^ Our lack of significant associations between sαKl levels and these biomarkers could imply that sαKl protein, unlike *KL*-VSHET genotype, does not modulate these non-specific AD pathophysiology markers, or that its influence may differ based on disease stage and cohort characteristics. The absence of associations in our sample, which included a range of AD stages, could also suggest that any influence of sαKl on non-specific markers like NfL, GFAP, and Ng is more detectable in preclinical or earlier stages of AD pathology, aligning with Driscoll et al.'s findings in cognitively unimpaired adults. These seemingly divergent findings highlight the complexity of sαKl ‘s involvement in AD and the potential influence of factors like sample characteristics and disease stage. However, without longitudinal data and larger sample sizes, drawing definitive conclusions remains challenging.

Finally, we explored the association between *KL*-VSHET and sαKl levels in a subset of participants with available data. Our results revealed significantly higher CSF sαKl levels in *KL*-VSHET carriers compared to those with the wild-type haplotype, consistent with prior studies reporting a relationship between *KL*-VSHET and increased CSF sαKl protein levels.^4,14^ However, consistently with Gaitán et al.,^14^ we did not find a significant difference in serum sαKl levels between the haplotype groups, which may be attributable to the limited sample size and potentially distinct dynamics of sαKl across different biological compartments. These findings suggest a potential haplotype-specific modulation of sαKl levels in the brain (as reflected by CSF measurements), which may not be captured by serum measurements.

Some study limitations should be noted. Our study was limited by sample size, which could have obscured potential associations. Future studies with larger samples are needed. Due to the small sample size, the *APOE* ε4 carrier group combined ε3/ε4 heterozygotes and ε4/ε4 homozygotes. This restricts our ability to determine if the observed associations between *KL*-VSHET and AD biomarker levels differ by *APOE* ε4 dose (one versus two ε4 alleles). Additionally, the cross-sectional design prevents definitive conclusions about directional relationships. Furthermore, α-Klotho protein analysis was not performed across both cohorts due to the limited availability of biological samples and data at the time of analysis. This prevented us from maximizing the sample size for these analyses. Finally, while we conducted a preliminary genotype-phenotype analysis on the subset of participants with both *KL*-VS haplotype and sαKl protein data, the limited sample size limits the strength and generalizability of these findings.

In summary, our key finding is that *KL*-VSHET carriers showed higher CSF Aβ_42_ and Aβ_42/40_ ratio levels that was attributable to differences in *APOE* ε4 carriers only. This suggests a potential protective effect of *KL*-VSHET against amyloid pathology, but only in individuals carrying the *APOE* ε4 allele. *KL*-VSHET haplotype did not show an association with measures of tau burden or neurodegeneration as measured by NfL, indicating that its protective effects may be more specific to amyloid pathways rather than neurodegenerative processes generally. In addition, there was no association observed between sαKl protein levels and AD biomarkers, nor with non-specific biomarkers of AD pathophysiology, including NfL, GFAP, and Ng. This lack of association suggests that sαKl protein may not directly influence these biomarkers or that its potential effects on neuroinflammation, neurodegeneration, and synaptic dysfunction markers may vary by disease stage and may be less detectable in advanced AD. Furthermore, the link between *KL*-VSHET and Aβ levels may not play a role in the context of sαKl levels, as the observed effects of *KL*-VSHET on Aβ burden do not seem to be mediated by sαKl concentration alone. While *KL*-VSHET generally correlates with sαKl protein levels,^14–16^ it might not fully capture the functional protein abundance or its specific activity within different compartments like CSF and plasma or serum, potentially leading to divergent results. Conclusive evidence remains elusive and further research using larger, stage-specific cohorts and longitudinal data will be crucial to disentangle the complexities surrounding *KL*-VSHET's and sαKl's involvement in AD and its potential as a therapeutic target or prognostic marker.
